# 血小板无效输注诊断和治疗中国专家共识（2022年版）

**DOI:** 10.3760/cma.j.issn.0253-2727.2022.11.003

**Published:** 2022-11

**Authors:** 中华医学会血液学分会

血小板无效输注（platelet transfusion refractoriness, PTR）是指患者在连续2次或多次接受足够剂量的随机供者来源的ABO血型相合的血小板输注后，血小板计数未见有效增加。PTR是血液系统疾病患者，尤其是输血依赖患者的常见现象，其总体发生率为5％～34％[1]–[3]，可导致患者血小板需求和出血风险增加，住院时间延长，影响患者总体生存[4]。目前国内外对于PTR的诊疗尚无指南及共识，在参考国内外该领域相关研究进展后制定本共识，旨在为PTR的规范化诊疗提供参考。

一、病因和发病机制

PTR病因主要包括非免疫因素和免疫因素两大类，其中非免疫因素占PTR的80％～90％，免疫因素占10％～20％[5]，详见表1。

**表1:** 血小板无效输注（PTR）的病因

非免疫因素
发热
感染
弥散性血管内凝血（DIC）、出血
移植物抗宿主病（GVHD）
血栓性疾病
药物
脾功能亢进
血小板储存因素
免疫因素
同种异体抗体
人类白细胞抗原（HLA）Ⅰ抗体
人类血小板抗原（HPA）抗体
CD36抗体
血小板自身抗体
其他
药物诱导产生的抗体
ABO血型抗体

（一）非免疫因素

非免疫因素导致PTR主要通过血小板消耗及破坏增加引起血小板减少，主要包括以下因素：

1. 发热：发热是PTR的危险因素之一[3],[6]，发热患者体内产生的白细胞介素1（IL-1）、IL-6、肿瘤坏死因子等致热源水平增加，进而激活单核-巨噬细胞系统，导致血小板破坏增加。

2. 感染：病原体及其代谢产物免疫刺激作用、内毒素直接损害、网状内皮系统捕获等可导致血小板激活和消耗增加。另外，巨核细胞分化和成熟也会受到影响[7]–[8]。

3. 血栓：血小板沉积是血栓形成的重要环节，血栓形成过程中血小板聚集、活化和消耗增加，影响血小板输注效果。

4. 弥散性血管内凝血（DIC）：DIC以凝血因子大量消耗、微血栓形成和出血为主要特征。该过程中血小板激活并消耗，继发出血者，血小板丢失增加，共同导致PTR。

5. 出血：大量出血增加血小板消耗，且随后的紧急扩容可引起血液相对稀释，是导致PTR的原因之一。

6. 移植物抗宿主病（GVHD）：急性和慢性GVHD患者除存在微血管病变外，血循环中血小板相关免疫球蛋白水平也可增加，导致血小板在循环中清除的速度加快。

7. 药物：药物相关性血小板减少较为常见（如非甾体类抗炎药和化疗药物）。血液恶性肿瘤患者使用的大部分化疗药物可抑制骨髓，损害巨核细胞，诱导血小板凋亡。

8. 脾功能亢进与巨脾：脾脏是血小板的重要破坏场所，脾亢与巨脾导致血小板破坏增加。

9. 血小板储存因素：血小板在20～24°C持续震荡条件下储存时间不超过5 d，储存时间延长会导致血小板质量下降，影响输注效果。

（二）免疫因素

免疫因素主要包括：人类白细胞抗原（HLA）抗体、人类血小板抗原（HPA）抗体和CD36抗体所导致的同种异体免疫反应，多发生在有妊娠、器官移植和多次输血等情况的患者[2]，此外，还包括CD47抗体、CD38抗体等。

1. HLA-Ⅰ类抗体：HLA-Ⅰ类抗体是PTR最主要的免疫介导因素，占免疫因素的80％～90％[9]。HLA是人类主要组织相容性复合体（MHC）基因簇的编码产物，分为Ⅰ类和Ⅱ类抗原，血小板表面主要表达Ⅰ类抗原，包括HLA-A、B、C位点抗原，其中PTR发生主要受HLA-A、B位点影响。HLA具有高度免疫原性，当患者初次输注血小板时，机体通过直接和间接两种途径识别异体HLA抗原，激活记忆性T细胞，当机体再次接触该抗原时，致敏T细胞迅速启动免疫应答，激活B细胞，产生抗体，表达相应HLA抗原的血小板则会被清除[2]。

2. HPA抗体：HPA是血小板特有抗原，定位于血小板膜糖蛋白（glycoprotein）复合物上，去白细胞不影响其产生。HPA抗原变异性低于HLA，因此介导同种异体免疫的发生率较低，为2％～11％[10]–[11]。HPA抗体单独致PTR较少见，常和HLA抗体共同作用[12]。

3. CD36抗体：血小板表面CD36又称GPⅣ，能促进血小板聚集和黏附，缺失影响血小板功能。CD36抗原缺失者接受输血、器官移植、妊娠、流产等刺激后机体产生CD36同种抗体，再次输注CD36抗原阳性血小板可导致PTR[13]。

4. 血小板自身抗体：可见于自身免疫系统紊乱等导致的疾病，其血小板相关免疫球蛋白非特异地与血小板表面抗原结合，导致血小板易被单核巨噬系统等清除，显著减少输注血小板的存活期。

5. 其他：部分药物也可通过诱导产生半抗原介导的抗体、“奎宁型”抗体、药物特异性抗体、纤维蛋白原受体拮抗剂依赖性抗体、非特异性自身抗体和免疫复合物等多种免疫途径介导PTR的发生[14]。此外，血小板表面也表达或从血浆中吸附少量ABO血型抗原，成为发生PTR的潜在原因。

二、诊断

（一）PTR诊断标准

血小板输注效果可综合实验室指标和临床效果来判断，目前主要有三个指标来量化评估血小板输注效果，包括输注后增量（PI）、血小板恢复率（PPR）和血小板校正计数增量（CCI）。具体计算公式及诊断标准见表2。

**表2:** 血小板无效输注（PTR）的诊断标准

指标	公式	PTR评估
输注后增量（PI）	输注后PLT−输注前PLT（×10^9^/L）	1 h PI<5×10^9^/L或24 h PI<10×10^9^/L
血小板恢复率（PPR）	PI× BV× PD^−1^（10^11^）	1 h PPR<30%或20~24 h PPR<20%
血小板校正计数增量（CCI）	PI× BSA（m^2^）×PD^−1^（10^11^）	0.1~1 h CCI<7.5×10^9^/L或20~24 h CCI<4.5×10^9^/L

注 BV：血容量，可参考Nadler公式[17]计算；BSA：体表面积，参考许文生氏公式[18]计算；PD：输注血小板量，一般每10 U或每治疗量单采血小板的血小板数量≥2.5×10^11^

当患者体表面积和具体的血小板输注量未知时，PI可作为临床实践中较为简单的评估依据。PPR、CCI的计算纳入了患者血容量及体表面积，可有效减少个体差异的影响，更精确地评估血小板的输注效果。推荐PTR评估标准为连续两次输注ABO血型相合的新鲜血小板后20～24 h CCI<4.5×10^9^/L和（或）PPR<20％[2],[7],[15]–[16]。

（二）血小板抗体初筛

怀疑PTR的患者，建议通过交叉匹配进行血小板抗体筛查。目前常用的血小板抗体检测方法主要包括以下几种：淋巴细胞毒试验（LCT）多用于分析单独的HLA抗体；对于HLA及HPA混合抗体，一般采用酶联免疫吸附试验（ELISA）、被动混合血凝试验（PMHA）、固相凝集法等。其中固相凝集法在国内应用较为广泛，抗体阳性者考虑免疫性PTR，并可根据此结果提供交叉匹配相合的血小板输注，但本法不能区分HLA-Ⅰ类抗体和HPA抗体。

（三）HLA抗体检测

免疫性PTR大多为HLA-Ⅰ类抗体所致，有条件的单位可进行特异性HLA抗体检测。目前实验室主要采用以下三种方法检测HLA抗体：ELISA、流式细胞术（FCM）和免疫磁珠液相芯片（Luminex）技术[19]。其中，Luminex法具有较高的敏感性和特异性。各种方法的比较见表3。

**表3:** HLA抗体不同检测方法比较

检测方法	优点	缺点
酶联免疫吸附试验（ELISA）	无需分离细胞，可同时检出HLA-Ⅰ类和Ⅱ类抗体	操作繁琐且费时，检测通量小
流式细胞术（FCM）	可同时筛选出抗HLA-Ⅰ类和Ⅱ类抗体，敏感性较高	检测通量小，无法分辨抗体的特异性，可重复性和稳定性受技术人员的影响较大
免疫磁珠液相芯片技术（Luminex）	具有高敏感性、高特异性、高通量、操作简便、快速准确	需特殊设备，检验成本高

（四）HPA抗体和CD36抗体检测

当患者考虑免疫性PTR，但HLA抗体阴性或者输注HLA匹配的血小板效果不佳时，需考虑进行HPA抗体检测，目前认为GPⅡb/Ⅲa、GPⅠb/Ⅴ/Ⅸ、GPⅠa/Ⅱa、CD109等抗原具有免疫原性[20]，同时也需考虑CD36抗体的存在。检测方法包括ELISA、血小板单克隆特异性抗体固定试验（MAIPA）和血小板免疫荧光法（PIF）等[11]。

三、干预

首先要注意预防PTR的发生。预防手段包括治疗相关原发病，减少患者对血小板的输注需求，进一步需注意尽可能识别和避免容易导致PTR的危险因素。对于PTR高风险患者，尽量使用去白/辐照的血小板以及匹配/相容的血小板。对于诊断为PTR的患者，首先需评估病因，对于非免疫因素，需处理诱因，同时予以对症支持治疗；对于免疫性因素，建议及时完善血小板抗体的筛查、交叉配型以及HLA分型，选择相合或相容的血小板输注，根据需要采用合适的免疫治疗及止血治疗。

（一）处理非免疫因素

非免疫因素是PTR的主要原因，应结合患者的临床表现进行个体化治疗，处理原发病。包括积极抗感染、纠正DIC、减停可疑药物、及时控制移植后GVHD及血栓相关疾病。脾亢或巨脾引起血小板减少患者可考虑脾切除或者增加首次血小板输注量。

（二）选择合适来源的血小板

1. 去白细胞和辐照的血小板：有危险因素存在的患者应预防性输注去白和辐照的血小板，以减少同种免疫的发生。白细胞是HLA同种免疫的主要原因，因此在血小板采集或输注前过滤白细胞，能有效减少长期输注血小板患者的HLA同种免疫发生。辐照可通过灭活淋巴细胞减少血小板和白细胞表面的HLA抗原，既可降低血小板抗体的产生，又不影响血小板功能，但对体内已经存在的抗体无作用。

2. 交叉匹配的血小板：将患者血清与多个供者来源血小板进行交叉反应，阴性者即为交叉匹配相合血小板，此方法无需进行HLA分型，耗时短、检测便捷，对包括HLA、HPA等所有抗体类型导致的PTR均有效，可广泛开展[21]。但每次输注均需要进行交叉匹配，且异体免疫风险增加，影响HLA匹配血小板的供应[22]。

3. HLA匹配的血小板：对于HLA抗体导致的PTR，建议选择性输注与患者HLA完全匹配或者允许性错配的血小板，HLA匹配程度分可为A、BU、BX、C、D级[1],[7],[23]，具体见表4。其中A和BU级匹配血小板输注效果最好，优于交叉匹配，但需要有大量HLA分型明确的库存血小板。C级、D级匹配度较弱，几乎与随机供者来源血小板输注效果相似，该方法对HPA抗体导致的PTR无效[22]。另外，也可尝试HLA表位匹配的血小板。

**表4:** HLA匹配度分级

分级	描述
A	HLA-A和HLA-B 4个抗原均匹配
BU	供者的HLA-A和（或）B抗原为纯合子，不存在与受体不同的HLA-A或HLA-B抗原
BX	供者的HLA-A或HLA-B其中一个与受体不匹配，但在同一交叉反应组内，共享公共的抗原表位，属于允许性错配^a^
C	供者拥有1个以上在受者中不表达的抗原，且存在一个非交叉反应抗原。
D	存在两个及以上非交叉反应抗原

注 ^a^其中交叉反应组指因为大多数HLA抗体是针对HLA抗原的公共位点，因此基于交叉反应组的匹配属允许性错配

4. HLA相容血小板：该种方法仅要求根据患者的特异性抗体，选择缺乏相应抗原的供者，只需检测患者HLA抗体，增加了合适供者范围[8],[22],[24]。部分HLA抗体存续时间短暂，需重复检测，以获得合适来源的血小板。

（三）免疫治疗

针对免疫因素导致的无效输注，若无匹配血小板，可选择合适的方法清除体内抗体和（或）抑制抗体产生。

1. 静脉输注免疫球蛋白（IVIG）：IVIG可通过封闭抗体而减少血小板破坏，对于伴有免疫异常的严重血小板减少患者有一定疗效。推荐剂量为0.4 g·kg^−1^·d^−1^，于输注血小板前静脉滴注[9],[25]–[26]。

2. 利妥昔单抗：利妥昔单抗能特异性地与B细胞表面跨膜抗原CD20结合，介导B细胞溶解的免疫反应，可清除体内B淋巴细胞，减少抗体的产生，可单次或多次使用[27]–[28]。排除相关禁忌证情况下，推荐HLA抗体阳性患者单次剂量375 mg/m^2^[29]–[30]。

3. 血浆置换：必要时使用新鲜冰冻血浆进行血浆置换[9],[31]。

4. 大剂量血小板输注：在无法获得匹配血小板的情况下，若合并严重出血，大剂量随机供者来源血小板输注可吸附血小板抗体，降低血小板输注需求[32]。

（四）其他治疗

严重出血时，如果无法获得HLA匹配的血小板，也可输注随机供者来源血小板。在排除用药禁忌和注意药物相关不良反应的前提下，可根据实际需要，合理使用以下治疗手段：

1. 抗纤溶药物：抗纤溶药物通过保护已形成的纤维蛋白凝块不被溶解而发挥止血作用，从而减少血小板输注，可作为PTR的附加治疗。常用氨基己酸和氨甲环酸。

2. 重组人凝血因子Ⅶa（rFⅦa）：rFⅦa可在血管损伤部位与组织因子结合通过促进凝血酶的生成发挥局部促凝止血作用。推荐剂量60～120 µg/kg，用药间隔6～12 h。rFⅦa可促进凝血酶生成、介导血小板快速活化，在血小板减少时也能发挥止血作用，既往有血栓病史及血栓形成倾向患者应进行凝血相关检查、评估血栓风险[33]–[35]。

3. 血小板生成素及其受体激动剂：可尝试使用促进巨核细胞分化、成熟及产板的药物，包括重组人血小板生成素、血小板生成素受体激动剂、IL-11等，以治疗血小板减少，促进血小板数量尽快恢复。

4. 脾切除术：在其他方法均无效并排除相关禁忌证的前提下，择期行脾切除术可减少血小板在脾脏中的破坏，从而改善PTR[36]。

四、结语

当前血小板输注存在供需矛盾，PTR受到临床工作者的极大重视，规范PTR的防治管理极为重要。在利用现有资源积极干预的同时，建议血液中心、输血科以及临床医生互相配合，建立HLA、HPA已知基因型供者库以及CD36抗原缺失档案库，方便患者尽快找到匹配的血小板。此外，本共识将根据PTR国内外高质量临床研究进展和实践经验不断完善。
